# Development and Evaluation of Topical Gabapentin Formulations

**DOI:** 10.3390/pharmaceutics9030031

**Published:** 2017-08-30

**Authors:** Christopher J. Martin, Natalie Alcock, Sarah Hiom, James C. Birchall

**Affiliations:** 1 St Mary’s Pharmaceutical Unit, Cardiff and Vale UHB, Cardiff CF14 4HY, UK; C.J.Martin@bath.ac.uk; 2 Cardiff School of Pharmacy and Pharmaceutical Sciences, Redwood Building, King Edward VII Avenue, Cardiff University, Cardiff CF10 3NB, UK; natalie.alcock101@gmail.com (N.A.); BirchallJC@cardiff.ac.uk (J.C.B.)

**Keywords:** gabapentin, topical, Carbopol^®^, Lipoderm^®^, human skin

## Abstract

Topical delivery of gabapentin is desirable to treat peripheral neuropathic pain conditions whilst avoiding systemic side effects. To date, reports of topical gabapentin delivery in vitro have been variable and dependent on the skin model employed, primarily involving rodent and porcine models. In this study a variety of topical gabapentin formulations were investigated, including Carbopol^®^ hydrogels containing various permeation enhancers, and a range of proprietary bases including a compounded Lipoderm^®^ formulation; furthermore microneedle facilitated delivery was used as a positive control. Critically, permeation of gabapentin across a human epidermal membrane in vitro was assessed using Franz-type diffusion cells. Subsequently this data was contextualised within the wider scope of the literature. Although reports of topical gabapentin delivery have been shown to vary, largely dependent upon the skin model used, this study demonstrated that 6% (*w*/*w*) gabapentin 0.75% (*w*/*w*) Carbopol^®^ hydrogels containing 5% (*w*/*w*) DMSO or 70% (*w*/*w*) ethanol and a compounded 10% (*w*/*w*) gabapentin Lipoderm^®^ formulation were able to facilitate permeation of the molecule across human skin. Further pre-clinical and clinical studies are required to investigate the topical delivery performance and pharmacodynamic actions of prospective formulations.

## 1. Introduction

Gabapentin is an anti-epileptic drug (AED) currently licensed for the treatment of partial epileptic seizures [1] and peripheral neuropathic pain (NP) conditions, such as vulvodynia, post-herpetic neuralgia and painful diabetic peripheral neuropathy [2]. Whilst gabapentin is considered to be better tolerated with fewer side effects than other AEDs, treatment of NP with oral gabapentin is still often limited by adverse effects [3], such as dizziness, somnolence [4], ataxia and fatigue [1]. Topical or localised drug delivery has been shown to limit the adverse effects of systemically delivered medications for NP whilst providing high concentrations of active at the site of administration [5]. Furthermore, a recent in vivo study has shown topically applied gabapentin to be efficacious in a diabetic rodent model of both allodynia and vulvodynia [6]. There is however no licensed topical product containing gabapentin available in the UK or, as far as the authors are aware, elsewhere. The product is available as a “pharmaceutical special” however, with reported efficacy when used as a treatment for peripheral NP [3,7].

Topical formulations aim to incorporate and deliver a drug substance into and across biological barriers, such as the skin and vaginal mucosa. Accordingly, there are many different types of vehicles used in topical cream, ointment and gel formulations. The latter may comprise a relatively simple hydrogel system utilising a synthetic polymer carbomer (e.g., Carbopol^®^). Indeed, the simplicity and versatility in formulating gel products has led to investigation of their use in the delivery of a diverse range of drug substances [8]. Additionally, many licensed products have utilised carbomer excipients within the formulation, including Doublebase^®^ gel, Ibuleve™ gel, Voltarol^®^ Emulgel^®^, GelTears^®^ and Viscotears^®^. In some instances, such as in the ocular preparations, the carbomer within the formulation is used for its hydrating and lubricating properties, but it is also used as a topical base where the aim is to deliver medicaments across skin, for example in non-steroidal anti-inflammatory gels [9]. Furthermore, complex formulations have also been investigated for the delivery of highly hydrophilic molecules. For example, Steluti et al. delivered 5-aminolevulinic acid from propylene glycol solutions containing glycerol monooleate [10], whilst Mbah and Nnadi successfully delivered gabapentin from both water-in-oil and oil-in-water microemulsions [11].

When topical products are formulated, a critical factor to consider is the ability of the drug molecule to enter and cross the skin, or other biological barrier, whether intended for local or systemic delivery. Typically, skin permeation studies employ a Franz-type diffusion cell assembly to investigate the potential of formulations to deliver substances through skin in vitro [10,11,12,13]. A variety of model permeation barriers are employed in such diffusion studies, ranging from synthetic membranes to biological membranes and tissues derived from animal and human skin. For example, in the aforementioned studies Arellano et al. [12] utilised excised full thickness rat skin whilst Mbah and Nnadi [11] used heat separated rat epidermis. Steluti et al. [10] utilised full thickness hairless mouse skin and Tas et al. [13] compared permeation across polyurethane membrane, full thickness rat skin and human epidermal membrane. These studies, amongst others, confirm that the permeation barrier is a critical parameter when assessing permeation characteristics. For example, Tas et al. [13] demonstrated statistically significant differences in penetration of the active substance across each of the barriers that were studied. Previous studies have also shown that the degree of hydration of the skin used as a permeation barrier can also significantly affect the permeation of molecules [14]. With direct relevance to this study, a number of recent publications have reported the delivery of gabapentin across skin [11,15,16,17]. However, these studies have involved a plethora of topical formulations and skin barriers, primarily including rodent and porcine models, which is reflected in the variable estimation of gabapentin permeation. 

The aim of the current study is to develop and optimise stable topical gabapentin formulations and to investigate their delivery capabilities using a human epidermal membrane model, which can be considered to have a close correlation to the human skin barrier found in vivo. Topical bases will include hydrogels and commercially available pre-formulated bases. Initially, ethanol will be used as a chemical permeation enhancer within the hydrogels, due to its long established use in topical formulations. However, dimethyl sulfoxide, dimethyl isosorbide, isopropyl myristate and propylene glycol will also be examined. Contextualisation of our findings within the wider scope of the literature will further the understanding of administering gabapentin topically for the effective and localised treatment of neuropathic pain, with associated reduction in systemic side effects.

## 2. Materials and Methods

### 2.1. Materials

Versatile™ cream, gabapentin, Carbopol^®^ 974P, methyl hydroxybenzoate, propyl hydroxybenzoate, sodium methyl hydroxybenzoate and sodium ethyl hydroxybenzoate were obtained from Fagron UK Ltd., Newcastle upon Tyne, UK.

Doublebase™ gel was purchased from Dermal Laboratories Ltd., Hertfordshire, UK.

All laboratory reagents were obtained from Fisher Scientific, Loughborough, UK unless otherwise stated. Phosphate-buffered saline (PBS) 0.01M, pH 7.4 was purchased from Sigma-Aldrich, Poole, UK. An oil-in-water (o/w) base (Lipoderm^®^) was obtained from PCCA (Professional Compounding Centers of America, Houston, TX, USA).

A compounded 10% (*w*/*w*) gabapentin in Lipoderm^®^ formulation was generously supplied by St Mary’s Pharmaceutical Unit, Cardiff, UK.

Full thickness human breast skin was obtained with ethical approval and patient consent (South East Wales Research Ethics Committee, Ref 08/WSE03/55, November 2008) from the Aneurin Bevan University Health Board, Newport, UK and University Hospital Llandough, Cardiff and Vale UHB, Cardiff, UK.

### 2.2. Methods

#### 2.2.1. Production of Topical Gabapentin Hydrogels

Initially, gabapentin was dissolved into the required mass of de-ionised water. Hydrogels were then processed as follows:

- For blank Carbopol^®^ and 0% ethanol (EtOH) gels, sodium methyl and ethyl hydroxybenzoate were also dissolved in de-ionised water.
- For hydrogels containing a permeation enhancer, methyl and propyl hydroxybenzoate were dissolved in the required mass of permeation enhancer solvent. Permeation enhancers included EtOH, dimethyl sulfoxide (DMSO), dimethyl isosorbide (DMI), isopropyl myristate (IPM) or propylene glycol (PG). The permeation enhancer mixture was added to the aqueous mixture and pre-mixed for 5 min.

Mixtures A or B were then transferred to a STD 1 Silverson^®^ mixer (Buckinghamshire, UK) fitted with a square hole high shear screen. Sufficient Carbopol^®^ powder was dispersed within the solution and mixed for approximately 30 min. Residual gel was removed from the working head and the formulation was allowed to stand at room temperature for approximately 1 h. Finally, a sufficient quantity of neutralising agent was added in a dropwise manner and mixed at low shear to cross-link each gel and provide appropriate viscosity.

#### 2.2.2. Production of Formulations Utilising Proprietary Bases

The appropriate quantity of gabapentin powder was triturated into one of the following bases and mixed by hand: Versatile™ cream, Doublebase™ gel or Lipoderm^®^ base. Each formulation was then made up to weight, to create a 10% (*w*/*w*) product, and mixed by hand or automated paddle until thoroughly mixed. Formulations were packaged into aluminium tubes and crimped for storage under ambient conditions prior to use. 

#### 2.2.3. Viscosity Measurement

Approximately 200 g of formulation was placed into a custom-made cylindrical plastic container. The formulations were then analysed using a Contraves Rheomat LG108 Viscometer. Each gel analysed was placed into a water bath set to 32 ± 2 °C and equilibrated for approximately 30 min before analysis. In all cases, the shear rate was varied in a stepwise manner up to a maximum of 64.7 s^−1^. 

#### 2.2.4. Formulation Release Studies

To determine release of gabapentin from the formulation, static Franz-type diffusion cells (D Jones, Loughborough, UK) were assembled by sandwiching 1.5 cm diameter discs of Whatman^®^ nitrocellulose membrane, pore size 0.2 µm, between matched donor and receptor chambers. The diffusion cells had a known receptor volume (mean volume 4.28 mL) and diffusional surface area (mean area 1.11 cm^2^). The two chambers were clamped together and the receptor compartment filled with degassed 0.01 M phosphate-buffered saline (PBS) pre-equilibrated to 37 °C. The cells were placed into a water bath set to 37 °C and approximately 1 g of formulation was applied into the donor chamber. Each formulation was gently stirred with a glass rod before the donor chamber was sealed with a section of Parafilm^®^ and a foil cap placed over the sampling arm. 200 µL samples were obtained from the cells at 0, 1, 2, 4, 20 and 24 h time points and frozen at −20 °C until analysis. 

#### 2.2.5. Preparation of Human Epidermal Membranes

Previously frozen full thickness human breast skin obtained from various female donors (aged 61–84 years), was defrosted for approximately 1 h at ambient temperature. Subcutaneous fat was removed by blunt dissection and each skin sample cut into sections of approximately 1.5 cm^2^. Sections were immersed in a water bath at 60 °C for 55 s before the epidermis was removed by forceps [18]. Epidermal membranes were wrapped in aluminium foil and frozen at −20 °C prior to use; all membranes were used within three months of preparation.

#### 2.2.6. Franz-Type Diffusion Cell Studies

“Non-hydrated” human epidermal membranes were defrosted for approximately 30 min at ambient temperature and assembled into static Franz-type diffusion cells with the stratum corneum (SC) facing the donor chamber. To create “hydrated” skin sections the membranes were assembled into the diffusion cells and pre-hydrated overnight with PBS (containing 0.138 M NaCl and 0.0027 M KCl) or 0.9% (0.154 M) sodium chloride solution (NaCl) in the receptor chamber. Diffusion cells had a known receptor volume (mean volume 3.50 mL) and diffusional surface area (mean area 1.13 cm^2^).

Prior to initiation of diffusion studies, donor and receptor chambers were filled with 0.9% NaCl, pre-heated to 37 °C, and placed into a water bath at 37 °C. Electrical resistance (ER) across each membrane was measured by passing a fixed current of 1 kHz across the skin using an Agilent U1731C Handheld LCR databridge connected to 2 stainless steel electrode probes. The negative electrode was inserted into the receptor chamber arm below the saline level, whilst the positive electrode was positioned in the saline contained in the donor chamber, taking care not to touch the membrane itself [19]. When required for control studies, 5 in-plane 750 µm long stainless steel microneedles (GeorgiaTech, Atlanta, GA, USA) were gently inserted into hydrated membranes twice and ER measurements were re-assessed prior to study initiation. 

Once initial ER measurements had been performed, donor and receptor media was removed from each of the cells and the receptor chambers were replaced with fresh, degassed 0.01 M PBS pre-equilibrated to 37 °C. The donor compartments of the diffusion cells were then loaded with approximately 1 g of formulation (representing infinite dose conditions). Each topical formulation was gently stirred before the donor chamber and sampling arms were covered. Samples were then obtained from the cells at 0, 1, 2, 4, 8 or 12 and 24 h and stored for analysis, as described in Section 2.2.4. Once final samples were taken at 24 h, the cells were emptied and re-filled with 0.9% NaCl before final ER measurements were taken.

#### 2.2.7. High Performance Liquid Chromatography (HPLC) Analysis

All samples were analysed by reversed-phase HPLC based on a validated method reported by Ciavarella et al. 2007 [20]. Briefly, separation was achieved on either a Pinnacle DB Cyano 5 µm or a Luna 5 µm CN 100 A column using an acetonitrile:10 mM phosphate buffer (8:92 *v*/*v*) (pH 6.2) mobile phase. Gabapentin was eluted isocratically at a flow rate of 1 or 1.5 mL/min respectively, and analysed with UV detection at 210 nm. Quantification of gabapentin in samples was performed using the calibration curve obtained from reference standard solutions dissolved in 0.01 M PBS. Standards were found to be linear in the concentration range 42–10,000 mcg/mL, with a limit of detection of 14 mcg/mL.

#### 2.2.8. Data Analysis

The apparent flux of gabapentin (*J*_ss(4–24 h)_) was calculated following the non-linear lag portion of the cumulative permeation data through a unit surface area of model membrane as a function of time using the following equation:

Flux (*J*_ss(4–24 h)_) = d(*Cr* × *Vr*)/dt/*A* (1)

where,

*Cr* is the cumulative receptor chamber concentration (mcg/mL).
*Vr* is the volume of the receptor chamber
*A* is the diffusional surface area of the membrane.

Where appropriate, a Student’s *t*-test was used to make direct comparisons between treatment groups. To make comparisons between multiple treatment groups a one-way ANOVA with Bonferroni’s multiple comparison post hoc test was performed. A *p*-value of less than 0.05 was considered to be statistically significant in all cases.

## 3. Results

### 3.1. Production of Topical Gabapentin Formulations 

The aqueous hydrogels produced in this study generally formed homogeneous, translucent to transparent formulations with a semi-solid nature. In comparison, the oil-in-water (o/w) formulations consisted of macroscopic emulsions that presented with a white to off-white colour. The organoleptic characteristics of all of the topical formulations and some viscosity and pH determinations of selected formulations are shown in Table 1.

**Figure 1:**
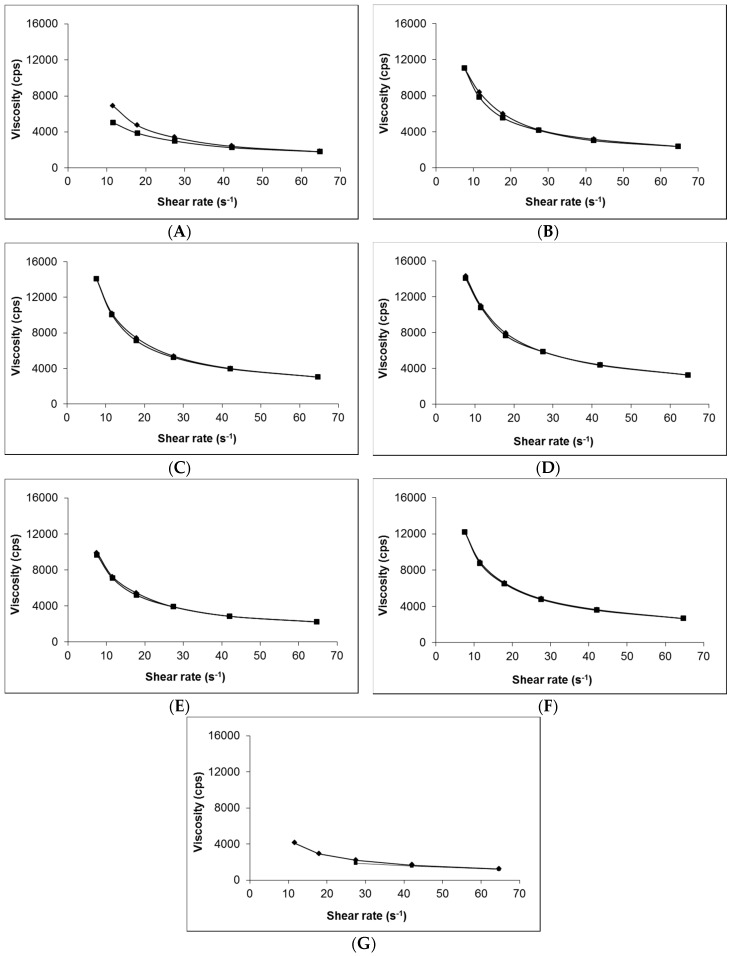
Viscosity and shear rate interrelationship of selected topical formulations. (**A**) blank Lipoderm^®^ base, (**B**) blank Carbopol^®^ 0.75% (*w*/*w*), (**C**) 6% (*w*/*w*) Carbopol^®^ 0.75% (*w*/*w*) gel containing 0% (*w*/*w*) EtOH, (**D**) 6% (*w*/*w*) Carbopol^®^ 0.75% (*w*/*w*) gel containing 30% (*w*/*w*) EtOH, (**E**) 6% (*w*/*w*) Carbopol^®^ 0.75% (*w*/*w*) gel containing 70% (*w*/*w*) EtOH, (**F**) 6% (*w*/*w*) Carbopol^®^ 0.75% (*w*/*w*) gel containing 5% (*w*/*w*) DMSO and (**G**) compounded 10% (*w*/*w*) Lipoderm^®^ formulation. ♦ represent increasing shear rate, ■ represent decreasing shear rate.

Figure 1 shows that, irrespective of composition (i.e., presence of gabapentin or penetration enhancer), the viscosity of each of the 0.75% (*w*/*w*) Carbopol^®^ hydrogels was very similar (Figure 1B–F). In contrast, the Lipoderm^®^ base (Figure 1A) had lower viscosity compared to the hydrogels and demonstrated hysteresis in its rheological behaviour; there was no hysteresis shown in the Carbopol^®^ gel formulations. The viscosity of the Lipoderm^®^ base was further decreased upon addition of 10% (*w*/*w*) gabapentin to the formulation (Figure 1G).

### 3.2. Release of Gabapentin from Topical Formulations

**Figure 2:**
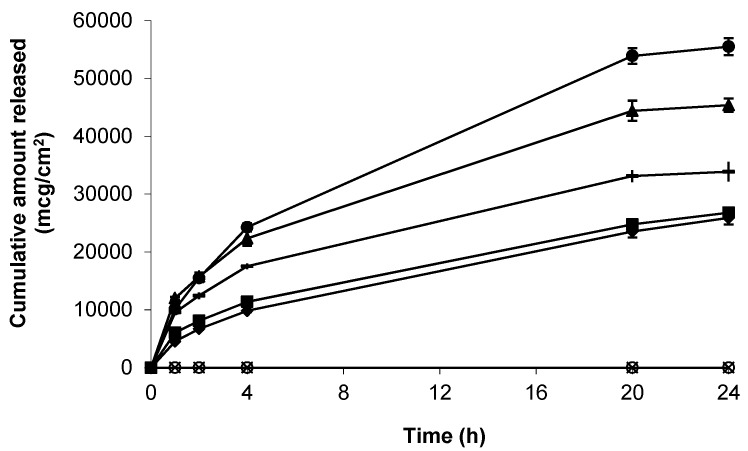
Cumulative release of gabapentin from the following topical formulations, 6% (*w*/*w*) Carbopol^®^ 0.75% (*w*/*w*) gel containing 5% (*w*/*w*) DMSO (**-**), compounded 10% (*w*/*w*) Lipoderm^®^ formulation (■), 6%*(w/w)* Carbopol^®^ 0.75% (*w*/*w*) gel containing 5% (*w*/*w*) IPM (▲), 6% (*w*/*w*) Carbopol^®^ 0.75% (*w*/*w*) gel containing 10% (*w*/*w*) DMI (●), 6% (*w*/*w*) Carbopol^®^ 0.75% (*w*/*w*) gel containing 70% (*w*/*w*) EtOH (♦), blank Lipoderm^®^ base (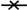) and blank Carbopol^®^ 1.5% (*w*/*w*) (○). Data presented as mean ± standard error of mean (SEM) (*n* = 3).

As a precursor to skin permeation studies, the release of gabapentin from each of the topical formulations was determined using a synthetic support membrane of porous, hydrophilic nitrocellulose. Release kinetics from all formulations was rapid over the first 4 h, and mean apparent flux values (mcg/cm^2^/h) were shown to be 4714.32 ± 227.33 (6% (*w*/*w*) Carbopol^®^ 0.75% (*w*/*w*) gel containing 10% (*w*/*w*) DMI) > 3440.63 ± 332.99 (6% (*w*/*w*) Carbopol^®^ 0.75% (*w*/*w*) gel containing 5% (*w*/*w*) IPM) > 2661.62 ± 50.39 (6% (*w*/*w*) Carbopol^®^ 0.75% (*w*/*w*) gel containing 5% (*w*/*w*) DMSO) > 1781.33 ± 38.21 (compounded 10%*(w/w)* Lipoderm^®^ formulation) > 1760.66 ± 82.06 (6%*(w/w)* Carbopol^®^ 0.75%*(w/w)* gel containing 70%*(w/w)* EtOH) (Figure 2).

### 3.3. Skin Permeation of Gabapentin from Saturated Hydroalcoholic Solutions

**Figure 3:**
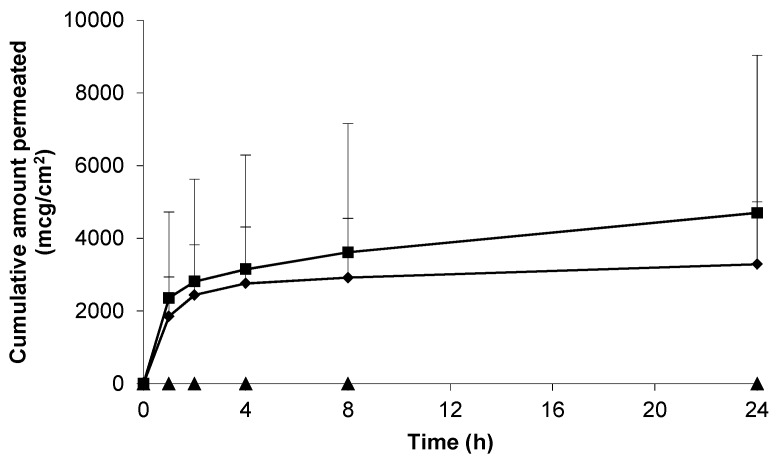
Cumulative permeation of gabapentin from a 50 mg/mL EtOH:H_2_0 (70:30) solution across pre-hydrated (♦) (*n* = 8) and non-hydrated (■) (*n* = 4) human epidermal membranes against control blank EtOH:H_2_0 (70:30) solution applied to pre-hydrated membranes (▲) (*n* = 2). Data presented as mean ± SEM.

Prior to assessment of the topical formulations the intrinsic skin permeation characteristics of the gabapentin molecule in saturated solution were assessed. Figure 3 shows that gabapentin permeated the human skin barrier from a saturated hydroalcoholic solution containing 70% (*w*/*w*) ethanol (EtOH); which correlated with previous findings involving a rodent skin model [11]. Furthermore, it has been previously speculated that the degree of membrane hydration may be a critical factor affecting the permeation of substances during skin diffusion studies [14,21]. However, this study showed that there was no significant difference in cumulative gabapentin flux between pre-hydrated (26.30 ± 11.00 mcg/cm^2^/h) and non-hydrated (77.56 ± 59.80 mcg/cm^2^/h) human skin membranes, *p* = 0.23 (summary shown in Table 2).

**Figure 4:**
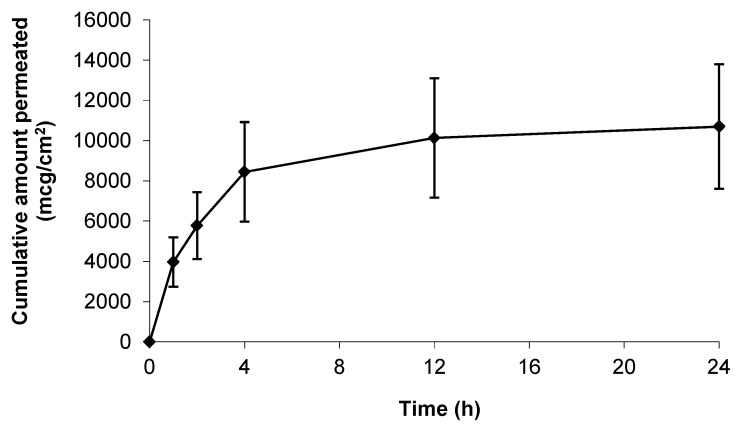
Cumulative permeation of gabapentin from a 50 mg/mL 70:30 EtOH:H_2_O solution following microneedle (MN) application to pre-hydrated human epidermal membrane. Data presented as mean ± SEM (*n* = 7).

As a positive control, and in an attempt to understand the upper limits of skin permeation of gabapentin, a solid microneedle (MN) array was applied to the epidermal membrane prior to application of the hydroalcoholic gabapentin solution. As Figure 4 shows, when the stratum corneum (SC) barrier was physically circumvented with MNs the permeation of gabapentin was approximately twice that observed in non-punctured skin (Figure 3).

### 3.4. Skin Permeation of Gabapentin from Topical Formulations

**Figure 5:**
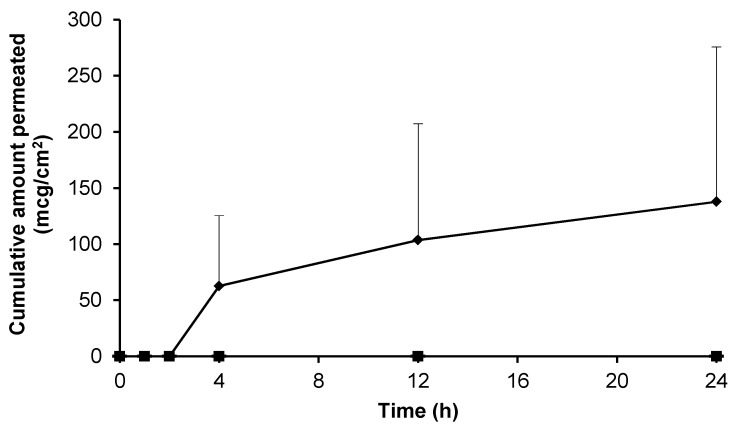
Cumulative permeation of gabapentin across pre-hydrated human epidermal membranes from the following hydrogels; 6% (*w*/*w*) Carbopol^®^ 0.75% (*w*/*w*) gel containing 70% (*w*/*w*) EtOH (♦, *n* = 3), 6% (*w*/*w*) Carbopol^®^ 0.75% (*w*/*w*) gel containing 30% (*w*/*w*) EtOH (■, *n* = 3), 6% (*w*/*w*) Carbopol^®^ 0.75% (*w*/*w*) gel containing 0% (*w*/*w*) EtOH (▲, *n* = 3), 10% (*w*/*w*) Carbopol^®^ 1.5% (*w*/*w*) gel (●, *n* = 4) and blank Carbopol^®^ 1.5% (*w*/*w*) gel (**-**, *n* = 8). Data presented as mean ± SEM.

The data presented in Figure 2 demonstrated effective gabapentin release from a variety of topical bases. The skin permeation characteristics of gabapentin released from each of the formulations was thereafter determined. Initially, Carbopol^®^ was used as a simple topical hydrogel containing no further excipients. The Carbopol^®^ 1.5%*(w/w)* gel containing 10%*(w/w)* gabapentin and no permeation enhancer failed to deliver the molecule through the SC skin barrier over 24 h (Figure 5).

Furthermore, it was found that Carbopol^®^ hydrogels containing 10%*(w/w)* drug became saturated and discrete crystals developed within the transparent gel matrix upon storage (data not shown). Therefore, in an attempt to optimise Carbopol^®^ based hydrogels, a range of Carbopol^®^ and gabapentin concentrations were investigated. Preliminary findings showed that gels containing 0.5% (*w*/*w*) Carbopol^®^ did not form gels of appropriate viscosity (data not shown), whereas gels containing 0.75% (*w*/*w*) Carbopol^®^ produced suitably viscous topical formulations. Additionally, the concentration of gabapentin within the gels was decreased to prevent crystallisation within the formulation. Consequently, the drug was incorporated at 6% (*w*/*w*) in a 0.75% (*w*/*w*) Carbopol^®^ hydrogel containing a permeation enhancer, as an optimised formulation. 

Initially, ethanol (EtOH) was incorporated within the formulation at a concentration of 30 or 70% (*w*/*w*) to act as a permeation enhancer. Of the hydrogels containing EtOH, quantifiable permeation of gabapentin was only observed following application of the 70% (*w*/*w*) EtOH gel (Figure 5). The apparent flux for this gel was 3.75 ± 3.75 mcg/cm^2^/h, indicating a large amount of variability. Furthermore, the permeation of gabapentin was also significantly lower than that observed in Figure 3, where a 70% hydroalcoholic solution was used as the donor vehicle. 

**Figure 6:**
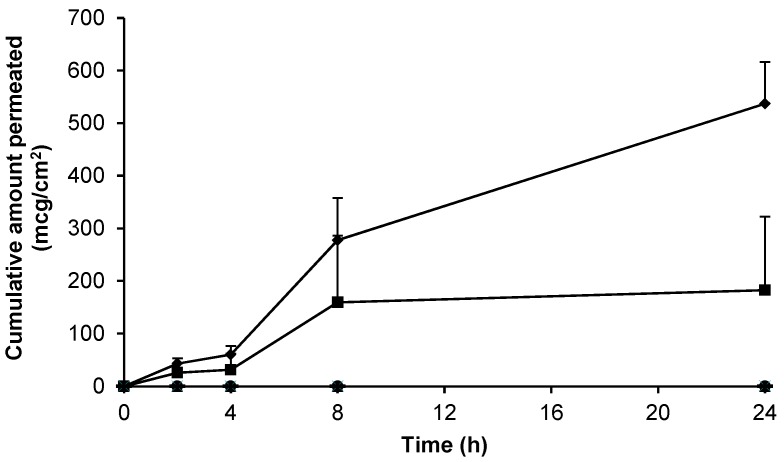
Cumulative permeation of gabapentin across human skin from 6% (*w*/*w*) Carbopol^®^ 0.75% (*w*/*w*) gel containing 5% (*w*/*w*) DMSO (■, *n* = 11), 6% (*w*/*w*) Carbopol^®^ 0.75% (*w*/*w*) gel containing 10% (*w*/*w*) DMI (▲, *n* = 3), 6% (*w*/*w*) Carbopol^®^ 0.75% (*w*/*w*) gel containing 5% (*w*/*w*) PG (●, *n* = 4), compounded 10% (*w*/*w*) Lipoderm^®^ formulation (♦, *n* = 18), blank Lipoderm^®^ base (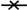, *n* = 3) and blank 0.75% (*w*/*w*) gel containing 5% (*w*/*w*) DMSO (-, *n* = 2) across non-hydrated human epidermal membrane. Data presented as mean ± SEM.

Although 70% (*w*/*w*) EtOH had been shown to facilitate gabapentin permeation from a Carbopol^®^ hydrogel across human skin, the enhancement was relatively small and variable (Figure 5). In an attempt to further improve skin permeation of gabapentin from topical preparations, Carbopol^®^ hydrogels containing other permeation enhancers and a compounded Lipoderm^®^ base were investigated as potential formulations (Figure 6). 

Figure 6 shows that hydrogels containing 6% (*w*/*w*) gabapentin and the permeation enhancers dimethyl isosorbide (DMI) or propylene glycol (PG) did not facilitate skin permeation of gabapentin from 0.75% (*w*/*w*) Carbopol^®^ gels. Furthermore, the commercial bases Versatile™ cream and Doublebase™ gel did not facilitate skin permeation of gabapentin (data not shown). Conversely, a Carbopol^®^ hydrogel containing 5% (*w*/*w*) DMSO and 6% (*w*/*w*) gabapentin, and a compounded Lipoderm^®^ formulation containing 10% (*w*/*w*) gabapentin, were shown to deliver the active substance across the skin barrier; with apparent flux values of 7.56 ± 5.50 mcg/cm^2^/h and 23.82 ± 3.51 mcg/cm^2^/h, respectively. In contrast to the saturated hydroalcoholic solution (Figure 3), a longer lag phase was observed with delivery from these formulations; however, there was no significant difference in gabapentin delivery between the two formulations (*p* = 0.08). Table 2 summarises the permeation results from this study in context with previously published studies of gabapentin skin permeation.

### 3.5. Electrical Resistance Measurement of Epidermal Membranes

**Figure 7:**
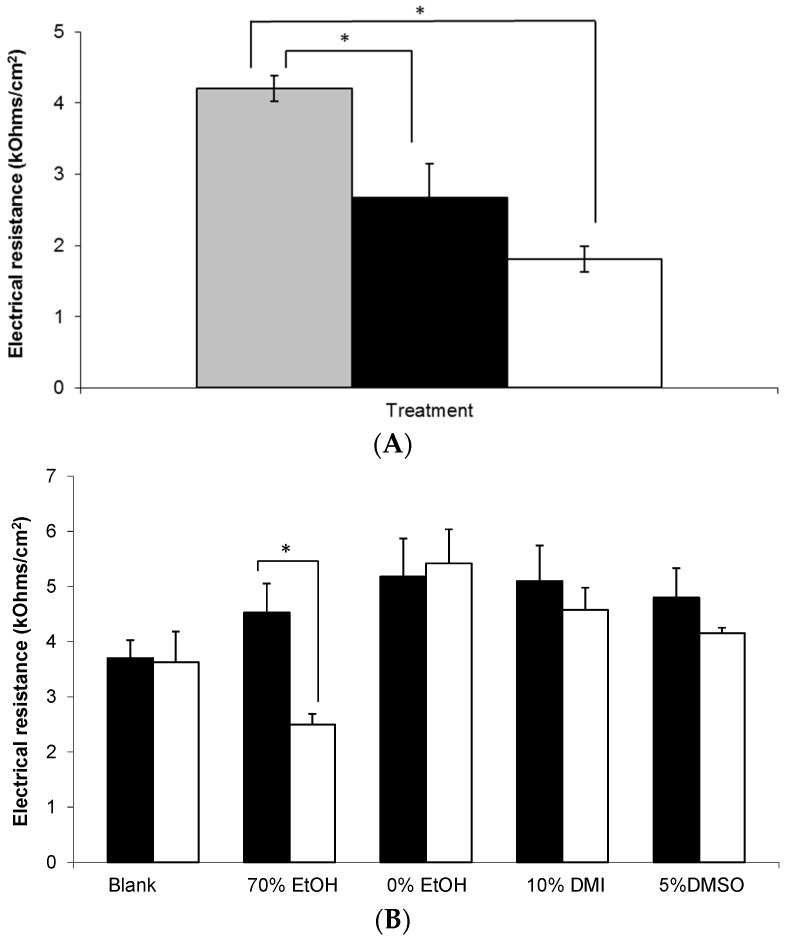
Electrical resistance measurement of human epidermal membranes. (**A**) Combined untreated skin at t = 0 h (grey bar) versus microneedle treated skin at t = 0 h (black bar) and t = 24 h (white bar), (*n* = 9–42); (**B**) Untreated skin at t = 0 h (black bars) and at t = 24 h (white bars) after application of the following treatments; (i) blank 0.75% (*w*/*w*) Carbopol^®^ gel, and 0.75% (*w*/*w*) Carbopol^®^ gels containing 6% (*w*/*w*) gabapentin and (ii) 70% (*w*/*w*) EtOH; (iii) 0% (*w*/*w*) EtOH; (iv) 10% (*w*/*w*) DMI; or (v) 5% (*w*/*w*) DMSO, (*n* = 3–5). Data presented as mean ± SEM, * represents *p* ≤ 0.05.

In order to assess the integrity of the skin barrier used in these studies, electrical resistance (ER) measurements were taken, as shown in Figure 7. Prior to application, the mean ER of all membranes treated with a topical gel was approximately 4 kOhms/cm^2^. Combined with detailed visual inspection of each membrane, this value was considered to be sufficient to demonstrate integrity of the SC barrier prior to study initiation. Subsequently, it was found that membranes treated with a MN array demonstrated significantly lower ER values at 0 and 24 h compared to untreated control skin, *p* < 0.05. However, there was no significant change in the ER of membranes treated with a MN array between 0 and 24 h (Figure 7A).

Figure 7B shows that whilst the majority of formulations did not affect the integrity of the membrane throughout the study period, there was a statistically significant decrease in ER at 24 h in the epidermal membranes treated with the 70% (*w*/*w*) EtOH Carbopol^®^ hydrogel (*p* = 0.041). 

## 4. Discussion

The aim of this work was to optimise a topical formulation to deliver gabapentin across the skin barrier. This approach is supported by in vivo studies [6] and observational clinical evidence [3,7], thereby providing a possible alternative treatment to oral dosing for neuropathic pain sufferers. Following pilot feasibility studies, two broad classes of formulation were identified as potential candidates; pre-formulated oil-in-water (*o*/*w*) bases and Carbopol^®^ hydrogels containing permeation enhancers. Carbopols^®^ are very high molecular weight polymers of acrylic acid which have traditionally been used as thickening and viscosity agents in liquid or semi-solid pharmaceutical preparations [23]. Once hydrated and neutralised, Carbopol^®^ polymers form an extended network with a mesh-like structure. Previously, these hydrogels have been shown to incorporate and release a range of drugs including low molecular weight compounds and macromolecules [18].

**Figure 8:**
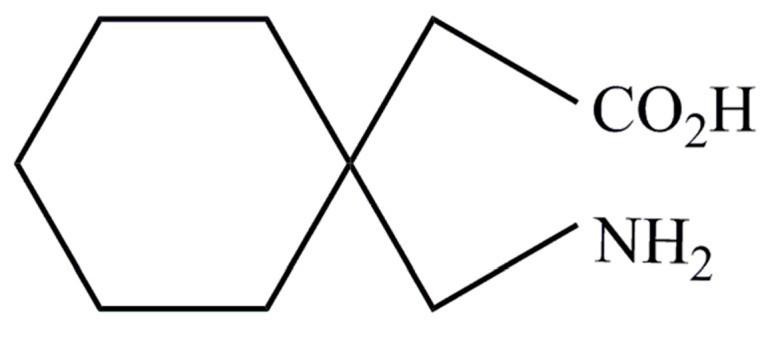
Gabapentin structure (reproduced from [1]).

Gabapentin is a low molecular weight, polar molecule (log *p* = −1.1) [24] which exists as a zwitterion at physiological pH [1] and demonstrates two pKa values; 3.68 and 10.70, respectively, for the carboxylic acid and primary amine group (Figure 8) [24].

It was found that Carbopol^®^ hydrogels containing 10% (*w*/*w*) drug became saturated and discrete crystals developed within the transparent gel matrix (data not shown). In support of this finding, other workers have also found that 7–10% gabapentin forms crystalline precipitations within topical formulations [15]. Subsequently, a maximum of 6% (*w*/*w*) gabapentin was incorporated within all Carbopol^®^ hydrogels to prevent gabapentin crystallisation within these systems. Furthermore, as gabapentin is a zwitterion, the pH within each of the formulations was optimised in an attempt to maintain drug stability. Previous studies have shown that the rate of gabapentin degradation in solution is minimal at a pH of approximately 6.0 and that the drug is most likely to exist as the zwitterionic species over the pH range of 4.5–7.0 [25]. Consequently, the pH of each formulation was developed to be within the range 6.0–7.0 to maintain gabapentin stability and ionisation state (Table 1). Once optimised, the three most promising candidate formulations, namely 0.75% (*w*/*w*) Carbopol^®^ gels containing 70% (*w*/*w*) ethanol (EtOH) or 5% (*w*/*w*) dimethyl sulphoxide (DMSO) and the compounded 10% (*w*/*w*) Lipoderm^®^ formulation were placed on stability trial under ICH storage conditions [26]. These trials demonstrated that the 5% (*w*/*w*) DMSO Carbopol^®^ gel and the 10% (*w*/*w*) Lipoderm^®^ formulation were stable, remaining within 90–110% stated content limits, under ambient conditions for a period of at least 3 months. However, the Carbopol^®^ gel containing 70% (*w*/*w*) EtOH was shown to be unstable, falling outside these limits, over the same time period when stored under these conditions (data not shown). 

Previous studies have demonstrated that hydrogels containing 1.0% (*w*/*w*) Carbopol^®^ are effective at releasing and delivering polar molecules across skin [13,27]. Consequently, a range of gels containing different concentrations of Carbopol^®^ were produced to investigate their rheological properties and topical drug delivery potential. However, this study showed that application of a Carbopol^®^ 1.5% (*w*/*w*) gel containing 10% (*w*/*w*) gabapentin to human skin membrane did not result in any skin permeation over 24 h (Figure 5). Even though gabapentin has a molecular weight of 171.2 [24], which suggests that it is likely to have a relatively large diffusion co-efficient [28], it was speculated that this negative finding may have been due to the hydrophilic nature of the molecule and precipitation of the active within the aqueous hydrogel, as stated previously. Furthermore, it was speculated that 1.5% (*w*/*w*) Carbopol^®^ may form a relatively dense gel matrix, thereby inhibiting gabapentin release. Subsequently, it was found that gels containing 0.5% (*w*/*w*) Carbopol^®^ did not form gels of appropriate viscosity (data not shown), whereas gels containing 0.75% (*w*/*w*) Carbopol^®^ produced suitably viscous topical formulations, and hence 0.75% (*w*/*w*) was used as the Carbopol^®^ concentration thereafter. Viscosity measurements were made at 32 ± 2 °C as previous studies have shown that the use of a water bath temperature of 37 °C for diffusion cell studies provides a skin (or skin membrane) surface temperature of 32 °C [29,30]. 

The measured viscosity of 0.75% (*w*/*w*) Carbopol^®^ gels containing 6% (*w*/*w*) gabapentin was in general agreement with other work describing the viscosity of various carbomer gels [23,31,32]. However, the viscosity of carbomer gels has been shown to vary significantly depending upon the specific form of polymer used and other excipients contained within the formulation [23]. Generally the viscosity of all formulations was shown to decrease with increasing shear rate, suggesting that the systems formed structured dispersions that were plastic in nature [32]. Previous studies have found that there is an inverse relationship between gel viscosity and drug delivery from the formulation [13]. Hence, it is noteworthy that even 0.75% (*w*/*w*) gels, containing the lowest feasible concentration of Carbopol^®^, retained approximately twice the viscosity of Lipoderm^®^ formulations. This may therefore have contributed to the relatively poor delivery of gabapentin across skin from the Carbopol^®^ based gels. Nevertheless, these findings provide further support for the approach to minimise the concentration of Carbopol^®^ contained within hydrogel formulations. 

Previously it has been shown that skin hydration can significantly affect stratum corneum (SC) properties and permeation of molecules across human skin [14,21]. However, in contrast, our study suggests that there was little difference in gabapentin permeability between membranes that had been pre-hydrated or not (Figure 3); although it did appear that there was less variability in permeation across pre-hydrated membranes. This finding may have been due to the polar nature of the gabapentin molecule, suggesting that it permeated a hydrated SC more reproducibly than across a non-hydrated epidermal membrane. Consequently, hydrated epidermal membranes were employed in all subsequent permeation studies. Our data suggests that gabapentin can permeate the human SC if applied to the skin in a vehicle with sufficient thermodynamic activity. This data correlates with the findings of [11,15,16] (Table 2), all of whom investigated skin permeation using in vitro porcine or rodent skin models. Furthermore, previous studies [15] have shown that gabapentin can be delivered across skin from simple aqueous solutions; although interestingly this is not supported by the work of [16].

In an attempt to enhance gabapentin skin permeation from topical formulations, permeation enhancers were incorporated within Carbopol^®^ hydrogels. Initially, EtOH was investigated due to its established use as a permeation enhancer in both licensed topical medicaments and within the scientific literature [27,29]; and due to early positive results shown in this work. Additionally, previous studies have shown that gabapentin in a solution of EtOH:water (70:30) permeates rodent skin [11]. In this work it was found that gabapentin permeates the human skin barrier from a hydroalcoholic solution containing 70% EtOH (Figure 3). It was speculated that the reason for this enhancement may have been due to partial extraction of lipids from the SC barrier [11,33] to facilitate gabapentin permeation. 

This study showed that 0.75% (*w*/*w*) Carbopol^®^ gels containing 6% (*w*/*w*) gabapentin in the absence of EtOH or incorporating 30% (*w*/*w*) EtOH (Figure 5), 5% (*w*/*w*) propylene glycol (PG) (Figure 6) or 10% (*w*/*w*) dimethyl isosorbide (DMI) (Figure 6) did not facilitate gabapentin permeation across human skin. Conversely, Carbopol^®^ gels containing 70% (*w*/*w*) EtOH (Figure 5), 5% (*w*/*w*) DMSO (Figure 6), and the compounded 10% (*w*/*w*) Lipoderm^®^ formulation (Figure 6) all facilitated gabapentin permeation over 24 h. Furthermore, whilst the formulation containing 70% (*w*/*w*) EtOH as a permeation enhancer showed only a relatively small and variable increase in gabapentin flux (3.75 ± 3.75 mcg/cm^2^/h), both the Carbopol^®^ hydrogel containing 5% (*w*/*w*) DMSO and the compounded 10% (*w*/*w*) Lipoderm^®^ formulation facilitated greater gabapentin permeation; with apparent flux values of 7.56 ± 5.50 and 23.82 ± 3.51 mcg/cm^2^/h, respectively. Although there was no significant difference in gabapentin apparent flux between these two formulations, the Lipoderm^®^ formulation delivered in a much more consistent manner. 

It has been stated that for small hydrophilic compounds, such as gabapentin, the applied vehicle controls the penetration pathway across skin [15], which may account for the variation in gabapentin delivery observed between the different formulations used in this study. Additionally, the effectiveness of DMSO to facilitate gabapentin permeation across human skin is supported by its use in topical formulations as a penetration enhancer [34,35] and studies showing that DMSO enhances permeation of various drug forms across a hairless guinea pig skin model to a greater extent than DMI [36]. However, it is also well known that DMSO, especially at higher concentrations, can be harmful to the body; acting both locally as a primary irritant, causing redness, burning, itching, scaling and urticaria and also systemically causing nausea, vomiting and ocular changes [37]. Lipoderm^®^ is not known to contain a toxic penetration enhancer, and hence it was hypothesised that this base may present a more clinically acceptable formulation. 

Although the sample sizes utilised in this study were reasonably large (Figure 6), extensive variability was observed within the permeation datasets. This variability was also observed by [17] who found large differences in the delivery of gabapentin from compounded Versatile™ cream across human trunk skin (Table 2). In contrast our gabapentin release data (drug release from the formulation rather than drug permeation) demonstrated considerably less variability. This serves to demonstrate the inherent variability of utilising a biological membrane for permeation studies and confirms that transfer of the drug across the biological barrier, rather than drug release from the formulation, will be the rate and dose limiting step in gabapentin delivery into and across skin. 

The apparent flux values obtained for the compounded 10% (*w*/*w*) Lipoderm^®^ and 5% (*w*/*w*) DMSO hydrogel formulations generally correlated with the values obtained from human skin sections treated with the control EtOH:water (70:30) saturated solution utilised in this study (Table 2). However, in relation to other studies discussed in this work, it is noteworthy that the skin model utilised in each experiment appears to affect the amount of gabapentin delivered. As Table 2 shows, the studies involving a rodent or porcine skin model [11,15,16] often demonstrated gabapentin flux values that were an order of magnitude greater than those studies involving human skin. The reason for the marked increases across rat skin may be due to the increased number of hair follicles found in rodent skin tissue [38] and hence an increased number of aqueous permeation pathways created in rodent epidermal skin sections following separation (of particular importance to a polar drug such as gabapentin). However, other works have suggested that for some drug molecules applied to dermatomed skin models, permeation across porcine and rodent skin provides a good correlation to human [39,40]. For example, Schmook et al. [40] investigated permeation of salicylic acid, a polar compound, across dermatomed human and porcine skin and full thickness rat skin and found a good correlation across all membranes. In contrast to the epidermal skin model, dermatomed skin is likely to retain a closer morphology to full thickness skin, which may account for the variability in drug permeation observed between these studies.

In the present study no gabapentin delivery was observed from a 10% (*w*/*w*) Versatile™ cream (Table 2), whereas Wang and Black [17] observed reasonable gabapentin permeation from the same formulation. Wang and Black [17] utilised ex vivo dermatomed human trunk skin as the model barrier and gently spread a finite dose over the skin surface. In this work an infinite dose was applied and only a very low spreading force was possible due to the extremely delicate nature of the epidermal membrane barrier. Consequently, it is speculated that the differences in skin model and experimental setup may account for the variation in gabapentin permeation shown between the two studies. 

This current paper describes the development of an in vitro human skin model using epidermal membranes with an intact electrical resistance (ER) measurement of approximately 4 kOhms/cm^2^ (Figure 7). Previously, it has been shown that an ER of 3.94 ± 0.37 kOhms/cm^2^ is representative of intact human epidermis [19] which is in good agreement with our findings. Figure 7 shows that there was a statistically significant decrease in ER at 24 h in the epidermal membranes treated with the 70% (*w*/*w*) EtOH hydrogel (Figure 7B). As gabapentin permeated across skin from this formulation, the associated drop in ER suggests that the high alcohol content may have disrupted the SC lipid barrier thereby facilitating gabapentin permeation. Other workers have used similar strategies to deliver actives from Carbopol^®^ hydrogels, for example, Pokharkar et al. [27] used an optimised vehicle containing 66.6% EtOH to facilitate zidovudine delivery. In a similar manner to this study, these authors found that drug permeation from gels not containing EtOH, or some other permeation enhancer, was very low in comparison [27]. Although there was a small decrease in ER observed in the membranes treated with the 5% DMSO gel (Figure 7B) the decrease was not shown to be significant. This potentially suggested an alternative mechanism of action for enhanced skin permeation by DMSO, such as displacement of bound protein water [41]. Accordingly, this mechanism would have a less marked effect on ER, which is predominantly a measure of SC lipid resistance to the passage of an applied electrical current [42]. 

As a positive control within this study, stainless steel microneedle (MN) arrays were inserted into selected epidermal sheets prior to application of hydroalcoholic gabapentin solution. Microneedle arrays contain a number of microscopic projections intended to be inserted into skin to penetrate the SC barrier without stimulating nerves or blood vessels found within deeper skin layers. Microneedle arrays have been shown to increase permeation of a range of different molecules across the skin barrier following pre-treatment including calcein [43], naltrexone, 5-aminolevulinic acid [44], insulin [45] and diphtheria toxoid adjuvanted with cholera toxin [46]. In this study, it was found that MN treatment significantly decreased the ER of epidermal membranes (Figure 7A). Although this finding was expected, ER has not been widely used as a measure of MN penetration efficacy, and hence this study suggests that ER may be employed as a measure of assessing MN insertion. Accordingly, Figure 7A suggested that MNs effectively punctured the SC to disrupt the lipid barrier, which correlated with the enhanced permeation of gabapentin observed (Figure 4). As expected, the MN-facilitated gabapentin permeation profile demonstrated no lag phase as the SC barrier was completely circumvented. However, it is not suggested that MN pre-treatment is likely to be a clinically viable approach for topical gabapentin delivery in this patient group due to the painful cutaneous symptoms experienced by many neuropathic pain sufferers. Indeed, our studies suggest that the use of an appropriate topical formulation can result in measurable quantities of gabapentin being effectively delivered without the need for physical disruption of the skin barrier. 

## 5. Conclusions

Topical delivery of gabapentin could provide an alternative treatment to oral delivery of the active for neuropathic pain conditions, with associated reduced systemic side effects; this is supported by in vivo studies and observational clinical evidence. However, gabapentin is a polar molecule and would not be expected to cross the stratum corneum barrier easily. In this study Carbopol^®^ hydrogels containing ethanol or dimethyl sulphoxide, and a compounded Lipoderm^®^ formulation, have demonstrated reasonable delivery of gabapentin across a biologically relevant in vitro human epidermal skin model. The compounded Lipoderm^®^ formulation emerged as the most consistent, stable and clinically relevant formulation. Whilst these findings are generally supported by other workers who have investigated topical gabapentin delivery, the results have been shown to vary from study to study dependent upon the skin model used and the formulation applied. Additional studies are required to explore the pharmacokinetic and pharmacodynamic responses to topically applied gabapentin in further detail.

## Figures and Tables

**Table 1:** Formulation components, characteristics and physico-chemical properties (of selected formulations).

Formulation					
Gabapentin (%(*w*/*w*))	Base (%(*w*/*w*))	Permeation Enhancer (%(*w*/*w*))	Characteristics	Viscosity (cps × 10^3^)	pH
0	Carbopol^®^ (1.5)	-	Transparent, homogeneous	-	-
0	Lipoderm^®^	-	Off-white, homogeneous	1.74	-
0	Carbopol^®^ (0.75)	-	Transparent, homogeneous	2.37	6.19
10	Carbopol^®^ (1.5)	-	Transparent, homogeneous	-	-
10	Lipoderm^®^	-	Off-white, homogeneous	1.20	6.00
10	Versatile™	-	Off-white, homogeneous	-	-
10	Doublebase™	-	White, homogeneous	-	-
6	Carbopol^®^ (0.75)	-	Transparent, homogeneous	2.98	6.34
6	Carbopol^®^ (0.75)	Ethanol (30.0)	Transparent, homogeneous, characteristic EtOH smell	3.28	6.65
6	Carbopol^®^ (0.75)	Ethanol (70.0)	Translucent, homogeneous, characteristic EtOH smell	2.24	7.15
6	Carbopol^®^ (0.75)	DMSO (5.0)	Transparent, homogeneous, characteristic DMSO smell	2.68	7.00
6	Carbopol^®^ (0.75)	IPM (5.0)	Transparent, biphasic	-	-
6	Carbopol^®^ (0.75)	DMI (10.0)	Transparent, homogeneous	-	-
6	Carbopol^®^ (0.75)	PG (5.0)	Transparent, homogeneous	-	-

**Table 2:** Permeation parameters of gabapentin from various vehicles across a number of different model skin barriers. *^a^*) Extrapolated from reference data.

Vehicle	Skin MODEL	Approximate Gabapentin Dose Applied (mg)	Mean J_ss(4–24 h)_ ± SEM (mcg/cm^2^/h)	Reference
5% (50 mg/mL) gabapentin in EtOH:H_2_0 (70:30) co-solvent solution	Human pre-hydrated breast skin epidermis	25	26.30 ± 11.00	This paper
5% (50 mg/mL) gabapentin in EtOH:H_2_0 (70:30) co-solvent solution	Human non-hydrated breast skin epidermis	25	77.56 ± 59.80	This paper
0.5% (5 mg/mL) gabapentin in EtOH:H_2_0 (70:30) co-solvent solution	Rat skin epidermis	5	63.29 ± 1.62	[11]
Gabapentin aqueous solution	Porcine skin	Unknown	Insignificant	[16]
Gabapentin water solution (100 mg/mL)	Dermatomed porcine skin	10	262.50	[15]
Compounded 10% (*w*/*w*) gabapentin in Lipoderm^®^ base	Human non-hydrated breast skin epidermis	100	23.82 ± 3.51	This paper
Combination 10% gabapentin in Lipoderm^®^ base	Dermatomed human trunk skin	0.5	0.11 *^a^*	[22]
10% (*w*/*w*) gabapentin 1.5% (*w*/*w*) Carbopol^®^ gel	Human non-hydrated breast skin epidermis	100	Insignificant	This paper
6% (*w*/*w*) gabapentin 70% (*w*/*w*) EtOH 0.75%*(w/w)* Carbopol^®^ gel	Human pre-hydrated breast skin epidermis	60	3.75 ± 3.75	This paper
6% (*w*/*w*) gabapentin 5% (*w*/*w*) DMSO 0.75% (*w*/*w*) Carbopol^®^ gel	Human non-hydrated breast skin epidermis	60	7.56 ± 5.50	This paper
6% (*w*/*w*) gabapentin 10% (*w*/*w*) DMI 0.75% (*w*/*w*) Carbopol^®^ gel	Human pre-hydrated breast skin epidermis	60	Insignificant	This paper
6% gabapentin 5% (*w*/*w*) PG 0.75% (*w*/*w*) Carbopol^®^ gel	Human non-hydrated breast skin epidermis	60	Insignificant	This paper
0.5% (5 mg/mL) gabapentin w/o microemulsion	Rat skin epidermis	5	128.22 ± 1.84	[11]
0.7% (6.9 mg/mL) gabapentin liposomes	Porcine skin	Unknown	219.90 ± 48.20	[16]
Gabapentin pluronic lecithin organogel	Porcine skin	Unknown	19.00 ± 10.60	[16]
Versatile™ cream (10% gabapentin)	Dermatomed human trunk skin	3	0.10 ± 0.10 *^a^*	[17]
Inverted hexagonal liquid crystals(2% gabapentin)	Dermatomed porcine skin	2	56.25 *^a^*	[15]
Lamellar liquid crystals (6% gabapentin)	Dermatomed porcine skin	6	92.31 *^a^*	[15]
10% (*w*/*w*) gabapentin in Versatile™ cream	Human non-hydrated breast skin epidermis	100	Insignificant	This paper
10% (*w*/*w*) gabapentin Doublebase™ cream	Human non-hydrated breast skin epidermis	100	Insignificant	This paper
